# Maturation of human pluripotent stem cell derived cardiomyocytes is improved in cardiovascular construct

**DOI:** 10.1007/s10616-017-0088-1

**Published:** 2017-04-10

**Authors:** Hanna Vuorenpää, Kirsi Penttinen, Tuula Heinonen, Mari Pekkanen-Mattila, Jertta-Riina Sarkanen, Timo Ylikomi, Katriina Aalto-Setälä

**Affiliations:** 1 FICAM, Finnish Centre for Alternative Methods, Faculty of Medicine and Life Sciences, University of Tampere, PL 100, 33014 Tampere, Finland; 2 BioMediTech, Institute of Biomedical Technology, Faculty of Medicine and Life Sciences, University of Tampere, PL 100, 33014 Tampere, Finland; 3 Faculty of Medicine and Life Sciences, University of Tampere, PL 100, 33014 Tampere, Finland; 4 Department of Cell Biology, Faculty of Medicine and Life Sciences, University of Tampere, Tampere, Finland; 5 Heart Hospital, Tampere University Hospital, Tampere, Finland

**Keywords:** Cardiomyocytes, Vascular-like network, Maturation

## Abstract

In order to translate preclinical data into the clinical studies, relevant in vitro models with structure and key functional properties similar to native human tissue should be used. In vitro cardiac models with vascular structures mimic the highly vascularized myocardium and provide interactions between endothelial cells, stromal cells and cardiomyocytes. Currently, human pluripotent stem cell-derived cardiomyocytes (hPSC-CMs) have been shown to present immature morphology and fetal-like electrophysiological properties that may limit their use as physiological test platform. The aim of this study was to develop multicellular in vitro cardiovascular construct modeling human heart tissue. In the cardiovascular construct, hPSC-CMs were cultured with a vascular-like network formed by human foreskin fibroblasts and human umbilical vein endothelial cells that served as a platform in the construct. Cardiomyocyte orientation, maturation, electrophysiological properties and drug responses of the cardiovascular construct were characterized and compared to CM monoculture. hPSC-CMs in cardiovascular construct showed elongated morphology and aligned with the vascular-like network. Electrophysiological properties and calcium metabolism of hPSC-CMs as well as response to E-4031 and adrenaline demonstrated normal physiological behavior. Increased expression of cardiac structural proteins and ion channels in cardiovascular construct compared to CM monoculture were detected. In conclusion, vascular-like network supports the structural and functional maturation of hPSC-CMs. Our results suggest that cardiovascular construct presents more mature in vitro cardiac model compared to CM monoculture and could therefore serve as an advanced test system for cardiac safety and efficacy assessment as well as a model system for biomedical research.

**Electronic supplementary material:**

The online version of this article (doi:10.1007/s10616-017-0088-1) contains supplementary material, which is available to authorized users.

## Introduction

In adult heart, the majority of cardiac cells are highly adaptive cells including fibroblasts, vascular smooth muscle cells and endothelial cells. 20–40% of the cardiac cells are cardiomyocytes (Soonpaa and Field 1998; Brutsaert 2003). In cardiac microenvironment, cardiomyocytes are embedded in aligned extracellular matrix (ECM) that facilitates the coordinated contractile function of the heart (Chien et al. 2008). Cells and ECM proteins, mainly produced by fibroblasts, are connected via cell–cell and cell–matrix interactions to maintain the structural organization and functionality of the heart (van Spreeuwel et al. 2014; Pfannkuche et al. 2010b). In mature myocardium, each cardiomyocyte has physical contact with at least one capillary blood vessel (Garzoni et al. 2009). The interactions between vasculature and myocardium are bidirectional (Bhattacharya et al. 2006), and active throughout the adult life affecting cardiac growth, function and rhythm (Brutsaert 2003). From the onset of the cardiac development, endothelial cells are the prerequisite for myocardial maturation, physiological function and survival (Brutsaert 2003). Endothelial cells and cardiomyocytes interact with each other with paracrine signals during heart development and activate the development of cardiac structure. This interaction is required for proper development of endocardium and myocardium. Vascular endothelial cells produce several compounds including angiopoietin 2 (Ang-2), whereas angiopoietin 1 (Ang-1) and vascular endothelial growth factor (VEGF) are mainly produced by cardiomyocytes (Brutsaert 2003; Leucker et al. 2011; Hsieh et al. 2006). In addition, fibroblasts secrete factors with autocrine and paracrine effects, such as VEGF, fibroblast growth factors (FGFs) and transforming growth factor beta (TGF-β), that give mechanical support and promote the organization of cardiomyocytes into 3D structures in collagen matrices (Pfannkuche et al. 2010b; Wong et al. 2007).

Cardiotoxicity is one of the leading causes of failure for a new therapeutic molecule preclinical development (Braam et al. 2010). Presently, pharmaceutical industry relies upon animal testing, although there are fundamental differences in the electrophysiological properties of animal and human cardiomyocytes (Feric and Radisic 2016). In particular, the differences in ion channels and currents impact the poor predictivity of drug screening and toxicity studies from murine to humans (Polini et al. 2014). In addition to animal models, transfected non-cardiac cells have been used in preclinical testing. However, these cells express usually only one cardiac ion channel and lack other characteristics of human cardiomyocytes. Transfected non-cardiac cells lack a cardiac intracellular environment that includes cardiomyocyte specific accessory proteins, as well as, cell structure (Martin et al. 2004). Therefore, there is an unmet need for improved human cardiomyocyte model for preclinical drug screening especially for assessment of cardiotoxic effects and for evaluation of the efficacy of new drug candidates (Feric and Radisic 2016; Kettenhofen and Bohlen 2008). The cell model should mimic effectively human cardiomyocytes for example in terms of cell structure, cardiac specific ion channels and electromechanical function.

Functional cardiomyocytes derived from human pluripotent stem cells could provide a significant advantage over the previously used test systems. Human pluripotent stem cell derived cardiomyocytes (hPSC-CMs) contract spontaneously and respond appropriately to cardioactive drugs (Robertson et al. 2013). These cells could be useful in early efficacy and toxicity screening, improving the selection of lead candidates and the reduction of adverse outcomes in clinical stages of drug development (Davila et al. 2004). According to their morphological and functional characteristics, hPSC-CM have been shown to display properties typical to human fetal cardiomyocytes which may complicate their utilization and interpretation of the obtained results (Snir et al. 2003; Robertson et al. 2013). Compared to the human adult cardiomyocytes the hPSC-CMs have been reported to have lower expression of the genes related to the ion transportation, calcium handling and sarcomere structure (Yang et al. 2014). Furthermore, the morphology of the hPSC-CMs does not resemble the well-aligned rod-like nature of adult human cardiomyocytes. Therefore, the maturation level of hPSC-CMs can be assessed by measuring the expression levels of genes encoding sarcomeric proteins such as cardiac Troponin I and T as well as α- and β myosin heavy chains. In addition, the expression levels of the cardiac specific ion channels and connexin 43 responsible for the intracellular communication can be used in estimation of the maturation state (Sartiani et al. 2007). In addition to the increased time in culture, electrical stimulation (Chan et al. 2013) and 3D culture environment (Schaaf et al. 2011; van Spreeuwel et al. 2014; Garzoni et al. 2009; Valarmathi et al. 2010; Pontes Soares et al. 2012) have been utilized in the production of more mature hPSC-CMs. However, the use of scaffolds to enable 3D environment has been associated with reduced cell–cell contacts, as well as incorrect deposition and alignment of extracellular matrix in cardiac constructs (Norotte et al. 2009).

In this study, a multicellular in vitro cardiovascular construct was developed. In the construct, hPSC-CMs were cultured with vascular-like network composed of human fibroblasts and human umbilical vein endothelial cells (HUVEC). Vascular-like network serves as a supporting platform to create natural microenvironment, i.e. biological scaffold, with cell–cell and cell–matrix interactions. The effects of the vascular-like network on cardiomyocyte morphology, gene expression and functionality were studied. Results demonstrated that the vascular-like network enhanced the structural maturation and increased the expression levels of cardiac ion channels in hPSC-CMs in the cardiovascular construct. Our study suggests that this cardiovascular construct provides a more mature in vitro cardiac model compared to CM monoculture and could therefore serve as an advanced test system for cardiac safety and efficacy assessment as well as a model system in biomedical research.

## Materials and methods

### Ethics

This study conforms to the principles outlined in the Declaration of Helsinki. The use of human umbilical vein endothelial cells (HUVEC) and induced pluripotent stem cells (iPSC) were approved by Ethics Committee of the Pirkanmaa Hospital District, Tampere, Finland (Approval Numbers R08028 and R08070, respectively) and a written informed consent was obtained from all the participants.

### Cell culture and differentiation

#### Isolation and culture of human umbilical vein endothelial cells

HUVECs were isolated from human umbilical cord vein by using enzymatic procedure as described previously by us (Sarkanen et al. 2011). Cells were detached from umbilical cords with 0.05% collagenase I, cultured in EGM-2 medium (Lonza Group Ltd, Basel, Switzerland, Table 1) and tested for mycoplasma contamination (MycoAlert^®^ Mycoplasma Detection Kit, Lonza Group Ltd) before experimental use. We use HUVEC as a standard source of endothelial cells in cell culture studies in our laboratory. The isolation and culture process of HUVECs has been established and validated producing cell lines with low variability (Sarkanen et al. 2011). Cells are routinely stored in liquid nitrogen in passage 2 and used for establishing cell culture models in passage 4. Each HUVEC line is derived from a single donor and quality tested routinely for their ability to form tubule structures in cell culture.

**Table 1:** Culture media used for development of cell models

Acronym	Basal medium	Serum	Growth factors	Supplementation
Fibroblast medium	MEM	10% FBS	–	1% l-glutamine, 1% NEAA
EGM-2 medium	EBM-2	2% FBS	VEGF, FGF-2, IGF, EGF	Hydrocortisone, ascorbic acid, heparin
Angiogenic stimulation medium	EBM-2	2% FBS	10 ng/ml VEGF, 1 ng/ml FGF-2	1% l-glutamine
EB 5%	DMEM/F12	5% FBS	–	1% NEAA, 1% Glutamax, 0.5% Pen/Strep

*VEGF* vascular endothelial growth factor, *FGF-2* fibroblast growth factor 2, *EGF* epidermal growth factor

#### Culture of human foreskin fibroblasts

Human foreskin fibroblasts were purchased from American Type Culture Collection (BJ, CRL-2522; ATCC, Manassas, VA, USA). Cells were cultured in fibroblast medium (Table 1) consisting of Minimum Essential Medium with Earle’s salts, w/o l-glutamine (Gibco, Vantaa, Finland) supplemented with 10% FBS (Gibco), 1% l-glutamine (Gibco) and 1% NEAA (Gibco). Cells were tested for mycoplasma contamination (MycoAlert^®^ Mycoplasma Detection Kit, Lonza) before experimental use.

#### Generation of patient-specific iPSC line and cell culture of pluripotent stem cells

In this study one commercial human embryonic stem cell (hESC) line H7 purchased from WiCell Research Institute (Madison, WI, USA) and one iPSC line was used for cardiomyocyte differentiation. Patient-specific iPSC line UTA.04602.WT was established from a healthy individual as described earlier (Takahashi et al. 2007). Shortly, skin biopsy from the donor was cultured in 0.2% gelatin (Sigma-Aldrich, Espoo, Finland) coated flask under fibroblast culturing conditions. iPSC line was established using lentivirus infection followed by retrovirus infection (Takahashi et al. 2007). Cells, plasmids and reagents used in this protocol include: 293FT cells, Plat-E cells, pLenti6/UbC/mSlc7a1-vector (Addgene, Cambridge, MA, USA), ViraPower™ Packaging Mix (Life Technologies Ltd), Lipofectamine™ 2000 (Life Technologies Ltd, Vantaa, Finland), Fugene 6 (Roche Diagnostics, Mannheim, Germany), and pMX retroviral vectors (hOCT3/4, hSOX2, hKLF4 and hc-MYC, all from Addgene). Results of the characterization of UTA.04602.WT cell line have been described earlier (Lahti et al. 2012).

UTA.04602.WT cells and H7 hESCs were cultured on mitomycin C inactivated mouse embryonic fibroblasts (MEF) in KSR medium which consisted of DMEM/F-12 (Invitrogen) supplemented with 20% KnockOut serum replacement (Invitrogen), 1% non-essential amino acids (Lonza), 2 mM Glutamax (Invitrogen), 50 U/ml penicillin/streptomycin (Lonza), 0.1 mM beta mercaptoethanol (Invitrogen) and 7.8 ng/ml basic fibroblast growth factor (R&D Systems). The medium was refreshed daily, and the stem cell colonies were passaged onto a new MEF layer once a week using 1 mg/ml collagenase IV (Invitrogen).

#### Differentiation of cardiomyocytes

Differentiation of pluripotent stem cells into cardiomyocytes was carried out with either by co-culturing hESC or iPSC with murine visceral endoderm-like (END-2) cells (Humbrecht Institute, Utrecht, The Netherlands) as described earlier (Mummery et al. 2003) or with small molecule differentiation method via temporal modulation of canonical Wnt signaling (Lian et al. 2013). Briefly, in END-2 method undifferentiated hPSC colonies were dissected mechanically into aggregates and plated on top of mitomycin C (Tocris) treated END-2 cells in the hPSC medium. The culture medium was supplemented with 2.92 mg/ml of ascorbic acid (Sigma-Aldrich). The medium was refreshed on days 5, 8 and 12. On day 14, 10% serum replacement was added to the medium. Briefly, for small molecule differentiation hPSCs maintained on a Geltrex-coated surface in mTeSR1 (Stemcell Technologies, Cologne, Germany) were dissociated into single cells with Accutase (Life Technologies) at 37 °C for 5 min and then seeded onto a Geltrex-coated cell culture dish at 50,000 cell/cm^2^ in mTeSR1 supplemented with 5 μM ROCK inhibitor (Tocris, Minneapolis, MN, USA) for 24 h. Cells were then cultured in mTeSR1, changed daily. On differentiation day 0, medium was exchanged with RPMI medium (Life Technologies) with 1× B27^®^ Supplement minus insulin (Life Technologies) with 10 μM CHIR99021 (Stemgent, Lexington, MA, USA). 24 h later, medium was exchanged with RPMI/B27 without insulin. On day 3, medium was exchanged with RPMI/B27 without insulin supplemented with 5 µM IWP4 (Miltenyi Biotech, Bergisch Gladbach, Germany). On day 5, medium was exchanged with RPMI/B27 without insulin. On day 7 and every 3 days following, medium was exchanged with RPMI/B27.

Both differentiation methods formed beating cardiomyocyte aggregates. These were mechanically excised and treated with collagenase A (Roche Diagnostics) to dissociate beating aggregates to single cell level (Mummery et al. 2003). In each experiment, cardiomyocytes plated on the monoculture and on top of the vascular-like network originated from the same differentiation and dissociation batch.

### Development of cell models

#### Human cardiomyocyte monoculture

After dissociation, human hPSC-CMs were seeded in EB 5% medium (Table 1) at density of 0.01–0.04 × 10^6^ cells/cm^2^ in 0.1% gelatin type A (Sigma-Aldrich) coated 48-well plates for immunocytochemical and qRT-PCR analyses and in microelectrode array (MEA) platforms and gelatin coated 12 mm cover slips for functional analyses. EB 5% medium was changed 1–2 days after cell seeding and thereafter twice a week. hPSC-CM monoculture in EB 5% medium was used as a control throughout the study.

#### Vascular-like network from co-culture of HUVEC and fibroblast

The co-culture was established as described earlier (Sarkanen et al. 2011). Briefly, fibroblasts (p 6–7), were seeded at 20,000 cells/cm^2^ in fibroblast medium (Table 1) in 48-well plates for immunocytochemical and qRT-PCR analyses and in MEA-platforms and 12 mm diameter cover slips for functional analyses and grown for 2–3 days to confluency. HUVEC were seeded on top of confluent fibroblast cultures at 4000 cells/cm^2^ in EGM-2 medium (Table 1). The day after cell seeding, angiogenic stimulation medium containing EBM-2 (Lonza), 2% FBS, 1 mM l-glutamine, 10 ng/ml vascular endothelial growth factor (VEGF, Sigma) and 1 ng/ml fibroblast growth factor 2 (FGF-2, Sigma) (Table 1) was applied to cells. The angiogenic stimulation medium was changed twice during the 6 day co-culture prior to CM seeding.

#### Cardiovascular construct

**Fig. 1:**
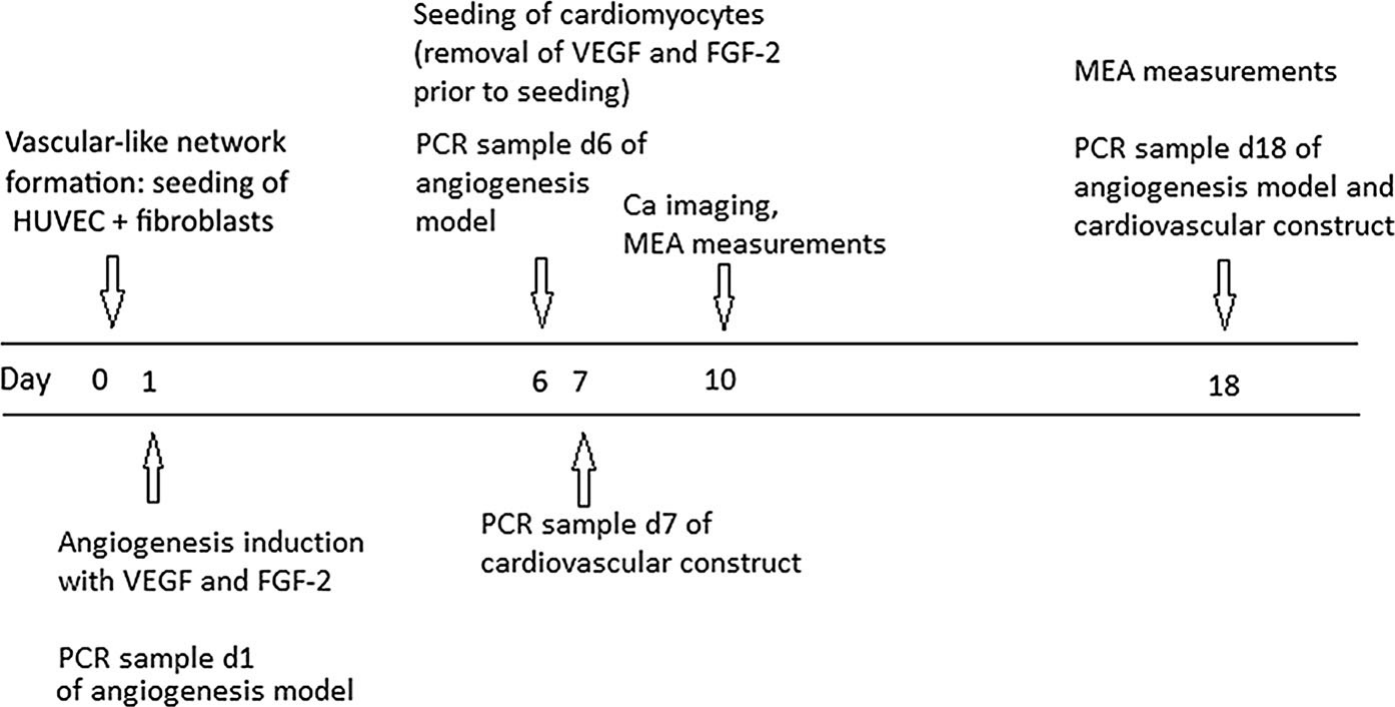
Establishment of cardiovascular construct

EB 5% medium (Table 1) was changed to HUVEC + fibroblast co-culture before seeding hPSC-CMs. Dissociated hiPSC- or hESC-derived cardiomyocytes in EB 5% medium (Fig. 1) were seeded on top of the HUVEC  + fibroblast co-culture at day 6, when the vascular-like network was already formed, at a density of 0.01–0.04 × 10^6^ cells/cm^2^ in 48-well plates for immunocytochemical and qRT-PCR analyses and in microelectrode array (MEA) platforms and 12 mm diameter cover slips for functional analyses. 1–2 days after cardiomyocyte seeding first EB medium change was performed and thereafter three times in a week. The viability of cardiovascular constructs was evaluated visually under microscope by assessing the contraction of the cardiomyocytes and vascular-like network formation of the HUVECs and fibroblasts at least three times in a week.

### Medium development

In cardiovascular construct, medium was designed to primarily support cardiomyocyte functionality and secondarily to induce vascular-like network formation. EB medium (Table 1) with different serum concentrations including 0, 2, 5, 10, and 20% FBS (Biosera-Immuno Diagnostic Oy, Hämeenlinna, Finland) were tested with duplicate wells for dissociated cardiomyocytes due to our objective to have low-serum or serum-free culture conditions, to avoid exogenous bias in our cell culture system and to develop in vitro model that mimics closely the in vivo situation in order to improve the predictive value of the test system. In that, it is important to provide physiologically relevant environment with low/no-serum culture conditions. Cells were cultured for 9 days and fixed with 4% PFA (Sigma-Aldrich) for immunocytochemical stainings with goat-anti-cardiac troponin-T (anti-Tnt, 1:1500, Abcam, Cambridge, U.K.). Cells were mounted with Vectashiel (Vector Laboratories, Burlingame, CA, USA) containing DAPI for staining nuclei. The results of the serum concentration tests can be found in the supplemental data (Fig. S1).

### Immunocytochemistry

Cardiomyocytes in CM monoculture and in cardiovascular construct were stained either with goat anti-cardiac-troponin-T (anti-Tnt, 1:1500, Abcam) or with mouse anti-Tnt (1:500, Abcam) and the vascular-like formation was visualized with basement membrane marker mouse collagen IV (anti-ColIV, 1:500, Sigma) for 1 h at RT or in + 4 °C o/n. Polyclonal IgG Alexa Fluor 568 (Abcam) for goat anti-Tnt and polyclonal IgG FITC (1:100, Sigma) for anti-ColIV and mouse anti-Tnt were used as secondary antibodies for 30 min in RT. Fluorescence was visualized with Nikon Eclipse Ti-S microscope (Nikon, Otawara, Tochigi, Japan) or with confocal laser scanning microscope Zeiss LSM780 Laser Scanning Confocal Microscope (ZEISS, Oberkochen, Germany). The images were processed with Zen2009 (confocal images, Carl Zeiss) and with Adobe Photoshop software 7.0 (Adobe Systems). The results are from at least 4 individual experiments performed in duplicates.

### Quantitative real time-PCR

Gene expression in CM monoculture, vascular-like network and cardiovascular construct were analyzed. The total RNA was extracted at day 1, 6, 7 and 18 (Fig. 1) using PureLink RNA Mini Kit (Life Technologies) following the manufacturer’s protocol. Concentration and purity of RNA was assessed using spectrophotometry with microplate reader in Varioskan Flash Spectrophotometer (ThermoScientific, Espoo, Finland) before further use. Reverse transcription of the total RNA to cDNA was performed using iScript cDNA synthesis kit (Bio-Rad, Helsinki, Finland) following manufacturer’s instructions. qRT-PCR was performed according to standard protocols on Abi Prism 7300 instrument (Applied Biosystems, Espoo, Finland) or on Bio-rad CFX96 Real Time System (BioRad). For each time point two biological replicate samples were collected and samples were analyzed as triplicates. The expression of angiogenesis related genes including *VEGF*-*A, FGF*-*2, PDGF*-*β, TGF*-*β1, Angiopoietin*-*1, Angiopoietin*-*2* and cardiac related genes including *CACNA1C, TNNT2, KCNJ2, CX43, MYH6, MYH7* were studied with SYBR chemistry. In addition, the expression of cardiac related genes *ADRB1* and *SCN5a* as well as reference gene *GAPDH* were studied using Taqman chemistry with Taqman Universal PCR Master Mix (Applied Biosystems). The following Taqman assays (20×) were used: Hs02330048_s1 for ADRB1, Hs00165693_m1 for SCN5A and Hs02758991_g1 for GAPDH (Applied Biosystems). SYBR primer sequences can be found in supplemental data (Supplementary Table 1). The relative expression levels were determined by using the comparative method (ΔΔCt) and GAPDH was used as an endogenous control (Livak and Schmittgen 2001, 402–408).

### Functional analyses

#### Microelectrode array (MEA) measurements

The ability of cardiovascular construct to conduct electrical signal was analyzed using the MEA system (Multi Channel Systems MCS GmbH, Reutlingen, Germany). The MEA platforms (8 × 8 standard MEAs or 6-well MEAs) were first hydrophilized with FBS and then coated with 0.1% gelatin type A (Sigma-Aldrich).

Field potentials were recorded at day 10 or 18 (Fig. 1) at 37 °C, and signals were recorded for 2 min. The sampling frequency was 20 kHz. Field potentials were recorded during spontaneous baseline beating, and with 1 µM adrenaline (Sigma-Aldrich) or 300 nM E-4031 (Sigma-Aldrich), which were incubated 2 min before measurements. Drugs were diluted and measurements performed in EB 5% medium. The field potentials were recorded with MC_Rack v.4.5.7 software (Multi Channel Systems MCS GmbH). Signals were analyzed with Cardiomyocyte MEA Data Analysis (CardioMDA) software (Pradhapan et al. 2013).

#### Analysis of Calcium^2+^ cycling

Cardiovascular constructs were loaded with 4 µmol/L Fura-2 AM (Invitrogen, Molecular Probes) for 30 min in HEPES based medium. Measurements were assessed in 37 °C and the extracellular solution consisted of (in mmol/L): 137 NaCl, 5 KCl, 0.44 KH_2_PO_4_, 20 HEPES, 4.2 NaHCO_3_, 5 d-glucose, 2 CaCl_2_, 1.2 MgCl_2_ and 1 Na-pyruvate (pH was adjusted to 7.4 with NaOH).

Ca^2+^ measurements were recorded at day 10 or 11 (Fig. 1) and conducted on an inverted IX70 microscope (Olympus Corporation, Vantaa, Finland) with a UApo/340 20× air objective (Olympus). Images were acquired with an ANDOR iXon 885 CCD camera (Andor Technology, Belfast, U.K.) synchronized with a Polychrome V light source by a real time DSP control unit and TILLvisION software (TILL Photonics, Gräfelfing, Germany). Fura-2 in cardiomyocytes was excited at 340 and 380 nm light and the emission was recorded at 505 nm. For Ca^2+^ analysis, regions of interests were selected for spontaneously beating cells and background noise was subtracted before further processing. The Ca^2+^ levels are presented as ratiometric values of F340/F380. The changes in Ca^2+^ were recorded during spontaneous baseline beating and spontaneous beating after 2-min 1 µM adrenaline (Sigma-Aldrich) perfusion.

### Statistics

qPCR data are expressed as mean values ±SD. Statistical analysis for the qPCR data was performed by IBM SPPS Statistics 22-software and by using Mann-Whitney *U*-test for independent samples. Bonferroni-correction was included when more than two groups were analyzed. A *p* value less than 0.05 was considered statistically significant.

In MEA and Ca^2+^ imaging data, the significance of differences between the two groups (CM monoculture and cardiovascular construct) was evaluated with the unpaired Student’s *t* test. The significance of changes within a group was evaluated with the paired Student’s *t* test. MEA and Ca^2+^ imaging data are expressed as average ± SEM. In Ca^2+^ imaging data n refers to the number of cells and in MEA data n refers to number of MEA wells. A *p* value less than 0.05 was considered statistically significant.

## Results

### Vascular-like network serves as a supporting and interactive platform for cardiovascular construct

**Fig. 2:**
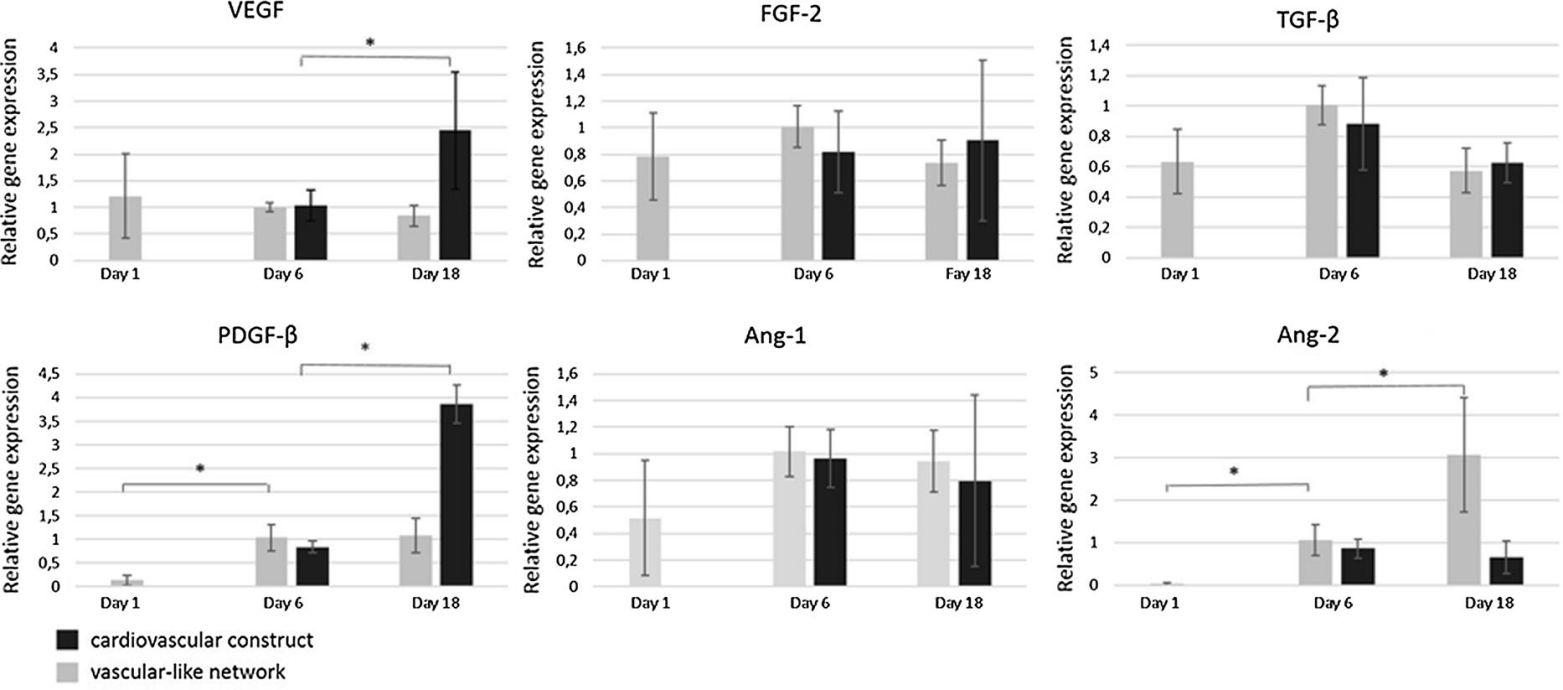
Relative gene expression levels of angiogenesis related genes including vascular endothelial growth factor (*VEGF*), fibroblast growth factor 2 (*FGF-2*), transforming growth factor beta (*TGF-β*), angiopoietin 1 (*Ang-1*), angiopoietin 2 (*Ang-2*) and platelet derived growth factor beta (*PDGF-β*) in cardiovascular construct and in vascular-like network at time points 1, 6 and 18. The *GAPDH* was used as a endogenous control. *p < 0.05

Expression levels of angiogenesis related genes were analyzed at days 1, 6 and 18 in vascular-like network to assess the production of growth factors and maturation state of vascular structures (Fig. 2). During the first six days the expression of *fibroblast growth factor 2* (*FGF*-*2), transforming growth factor β* (*TGF*-*β)* and *angiopoietin*-*1* (*Ang*-*1)* had an increasing trend whereas *VEGF* expression remained constant. Statistically significant increase was observed in *angiopoietin 2* (*Ang*-*2*) and *platelet derived growth factor β* (*PDGF*-*β*) expression. During the days 6–18, the expression of aforementioned genes remained constant except for *Ang*-*2*, which had significantly increased expression level.

The expression levels of the angiogenesis related genes were also assessed from the cardiovascular construct at day 6 and 18 (Fig. 2). The expression levels of *FGF*-*2*, *TGF*-*β* and *angiopoietin*-*1* remained quite constant during culture, whereas *Ang*-*2* expression was decreased in cardiovascular construct compared to the vascular-like network. However, the expression levels of *VEGF* and *PDGF*-*β* were significantly higher in the cardiovascular construct.

### Vascular-like network enhances mature cardiac phenotype and orientation of pluripotent stem cell derived cardiomyocytes in cardiovascular construct

**Fig. 3:**
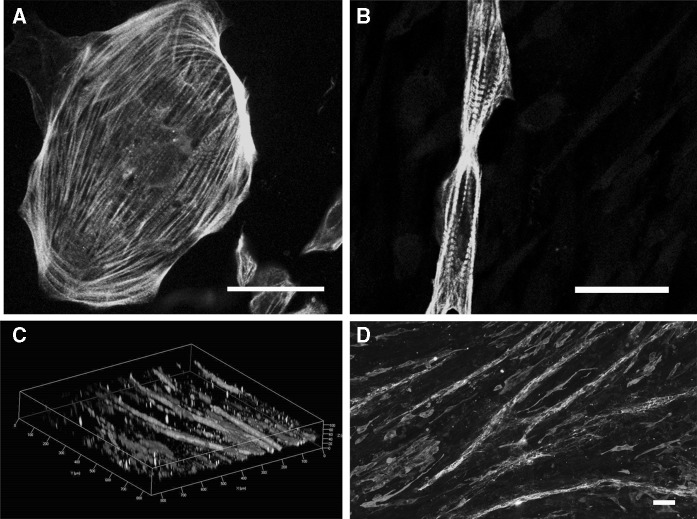
Morphology of human pluripotent stem cell derived cardiomyocytes in **a** monoculture and in **b** cardiovascular construct at day 14 in 10% FBS. Orientation of human pluripotent stem cell derived cardiomyocytes (troponin T, *red*) with vascular-like network (collagen IV, *green*) in **c**, **d** cardiovascular construct at day 7 in 5% FBS. **C** Confocal 3D z-stack projection having the *scale bars* included in the image. The vertical height is 100 µm and the horizontal length between two major tick intervals is also 100 µm. In **a**, **b** and **d** the *scale bars* represent 50 µm. (Color figure online)

Compared to the round morphology and the random orientation of cardiomyocytes in monoculture (Fig. 3a; S1), more mature morphology with elongated cardiomyocytes was detected in the presence of vascular-like network (Fig. 3b). Moreover, 2D and 3D projections showed that cardiomyocytes co-localized longitudinally and parallel with tubular structures (Fig. 3c, d). On the contrary, cardiomyocytes in monoculture remained rounded, less organized and the orientation of the cells along the tubule structures could not be detected.

**Fig. 4:**
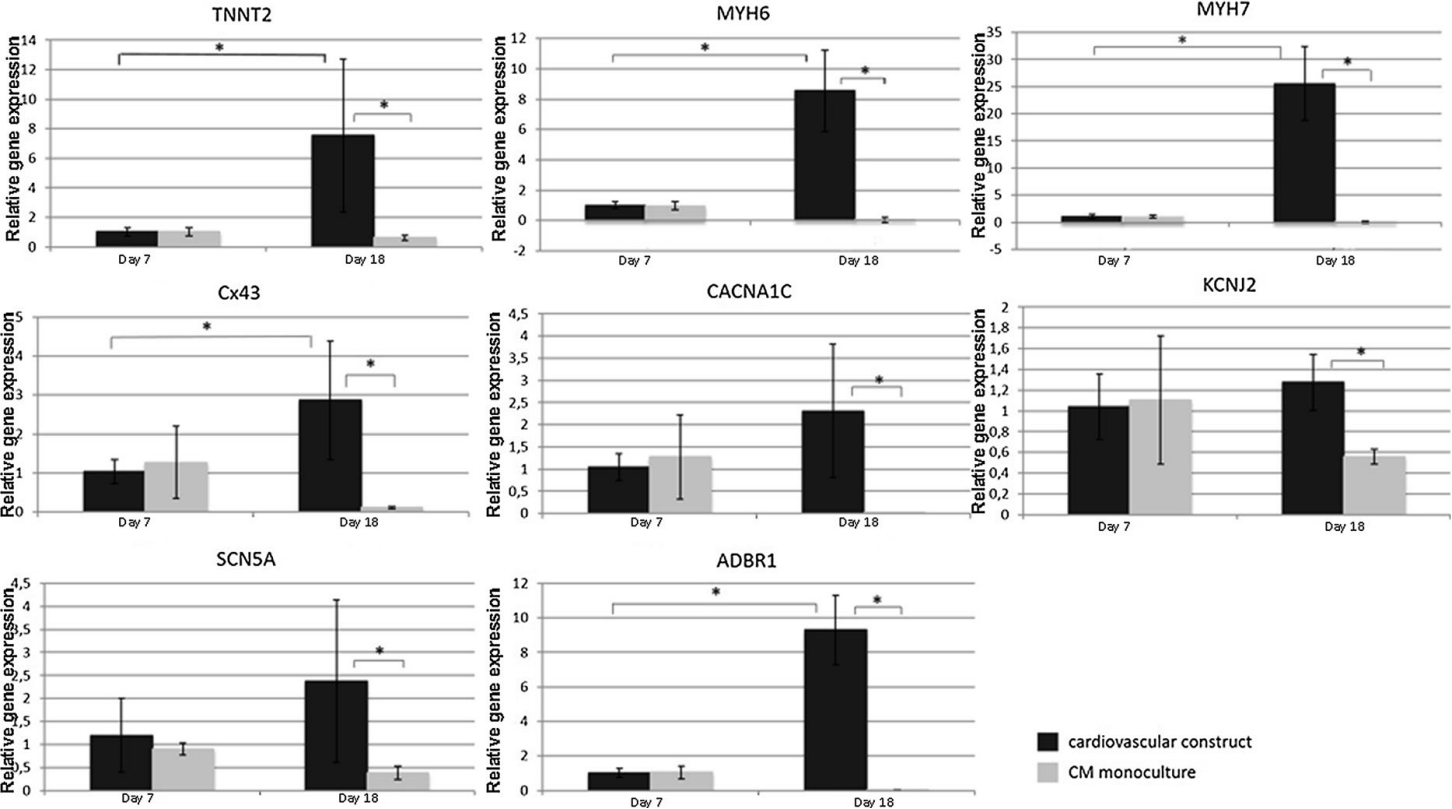
Relative gene expression levels of cardiac related genes troponin T (*TNNT2*), cardiac muscle myosins (*MYH6*, *MYH 7*), connexin 43 (*Cx43*), calcium channel (*CACNA1C*), potassium channel (*KCNJ2*), sodium channel (*SCN5A*) and beta-1-adrenergic receptor (*ADBR1*) at time points day 7 and day 18 in cardiomyocyte monoculture and in cardiovascular construct. The *GAPDH* was used as an endogenous control. *p < 0.05

The expression levels of cardiac related genes were analyzed in the cardiovascular construct and in the CM monoculture at one day after cardiomyocyte seeding (day 7) and at day 18 (Fig. 4). Results showed that the expression of cardiac muscle myosin transcripts *MYH6* and *MYH7* as well as cardiac *troponin T* increased between day 7 and 18 (*p* < 0.05) whereas the expression remained constant or decreased in the CM monoculture. The expression level of gap junction marker *connexin 43* was also shown to increase significantly in cardiovascular construct (*p* = 0.002) between day 7–18 while remaining low in the CM monoculture. At day 18 the expression levels of *MYH6, MYH7, troponin T* and *connexin 43* were all at higher level in the cardiovascular construct when compared to the CM monoculture (*p* < 0.05) (Fig. 4).

In the cardiovascular construct, the expression levels of ion channels *CACNA1C, KCNJ2* and *SCN5A* did not increase significantly between days 7–18 (Fig. 4). However, similarly to structural genes, there was statistically significant difference when expression levels were compared between cardiovascular construct and CM monoculture at day 18. The expression of *beta*-*1 adrenergic receptor* (*ADRB1*) was significantly higher at day 7 to day 18 in the cardiac construct (*p* > 0.05). Moreover, the expression was significantly higher at day 18 in the cardiovascular construct compared to CM monoculture.

### Electrophysiological changes in pluripotent stem cell derived cardiomyocytes in cardiovascular construct suggest functional improvement of the cardiomyocytes

**Fig. 5:**
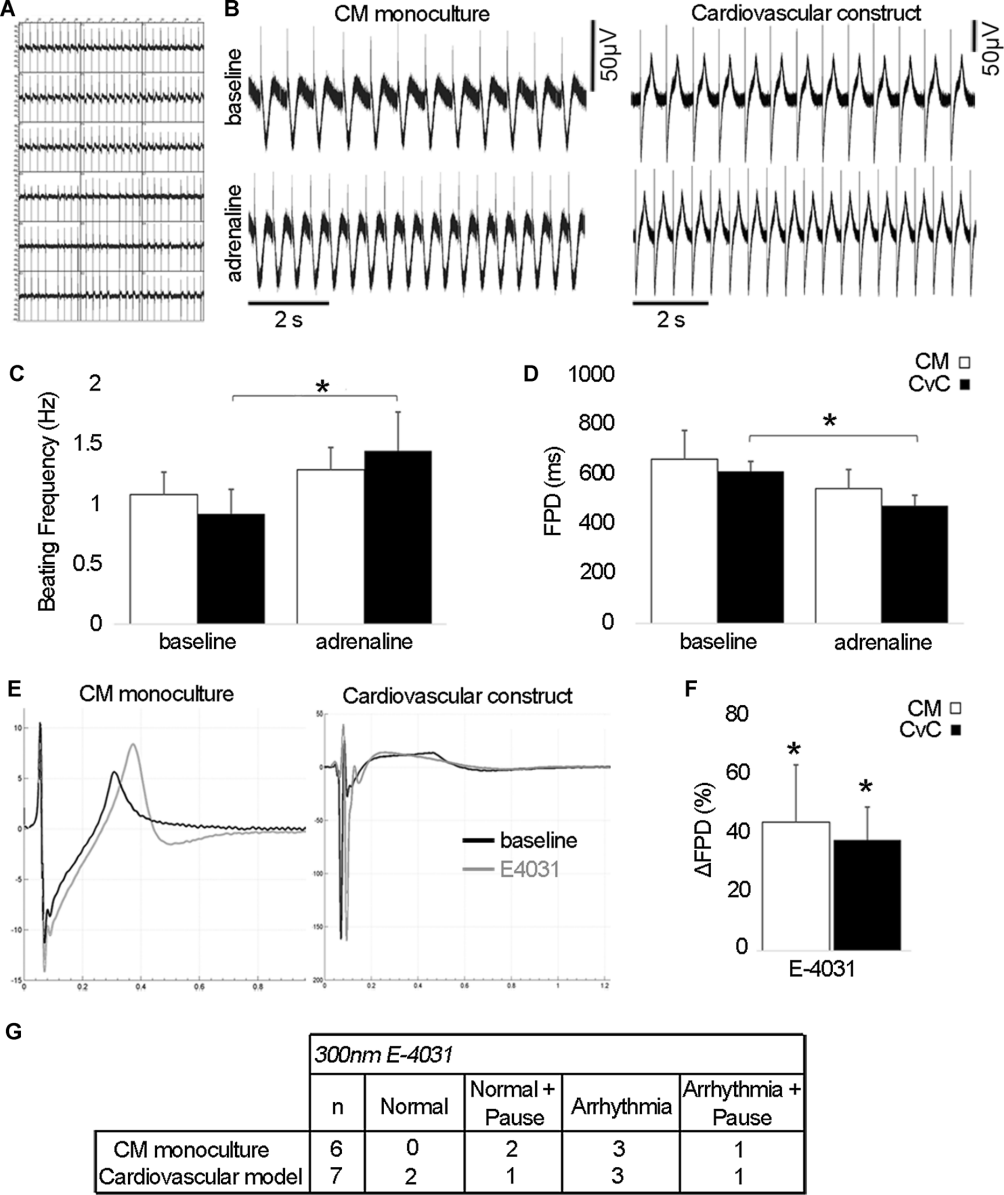
MEA measurements of the cardiovascular construct and CM monoculture. **a** The electrical activity of the cardiovascular construct was detectable with MEA and the contractility was synchronous; **b** representative field potential traces of the effect of β-adrenergic agonist adrenaline (1 μM); **c** beating frequency and **d** field potential duration (FPD) of baseline and adrenaline measurements of CM monoculture and cardiovascular construct (both n = 5). **e** Overlay plot showing the effect of 300 nM E-4031 on FPD; **f** the effect of E-4031 on FPD was calculated as relative change from the baseline in both CM monoculture (n = 6) and cardiovascular construct (n = 7). In both of these FPD was significantly prolonged with E4031 when compared to baseline but there was no significant difference between CM monoculture and cardiovascular construct. **g** Adverse effects of E-4031 during the time of 2 min measurement. The *number* indicates how many replicates were affected. Normal: no arrhythmias, Normal + Pause: no arrhythmias but field potential activity terminates, Arrhythmia: arrhythmias affecting amplitude and frequency, Arrhythmia + Pause: arrhythmias leading to termination of beating activity. *CM* cardiomyocyte monoculture, *CvC* cardiovascular construct. *p < 0.05

Contractility areas of the cardiovascular model were large and synchronous and beating was stronger when compared to moderately beating CM monoculture with smaller separately beating areas (Video S1, S2 and S3). The electrical activity of the cardiovascular construct was detectable with MEA and the contractility was synchronous (Fig. 5a). Adrenaline increased the beating frequency of cardiomyocytes (Fig. 5b, c) thus decreasing the field potential duration (FPD) in cardiovascular constructs as well as in CM monoculture (Fig. 5d). The increase in beating frequency and decrease in FPD was statistically significant between baseline and adrenaline only in cardiovascular construct (Fig. 5c, *p* < 0.05).

E4031, a hERG blocker, was shown to significantly prolong the FPD both in cardiovascular construct and in CM monoculture compared to the baseline (both *p* < 0.05) but there was no significant difference between the groups (Fig. 5e, f). Prolongation of FPD due to E4031 exposure resulted in additional adverse effects including pausing beating and/or arrhythmias. These adverse effects were seen in CM monoculture as well as in cardiovascular construct (Fig. 5g).

### Ca^2+^ transients of pluripotent stem cell derived cardiomyocytes in the absence and presence of vascular-like network

**Fig. 6:**
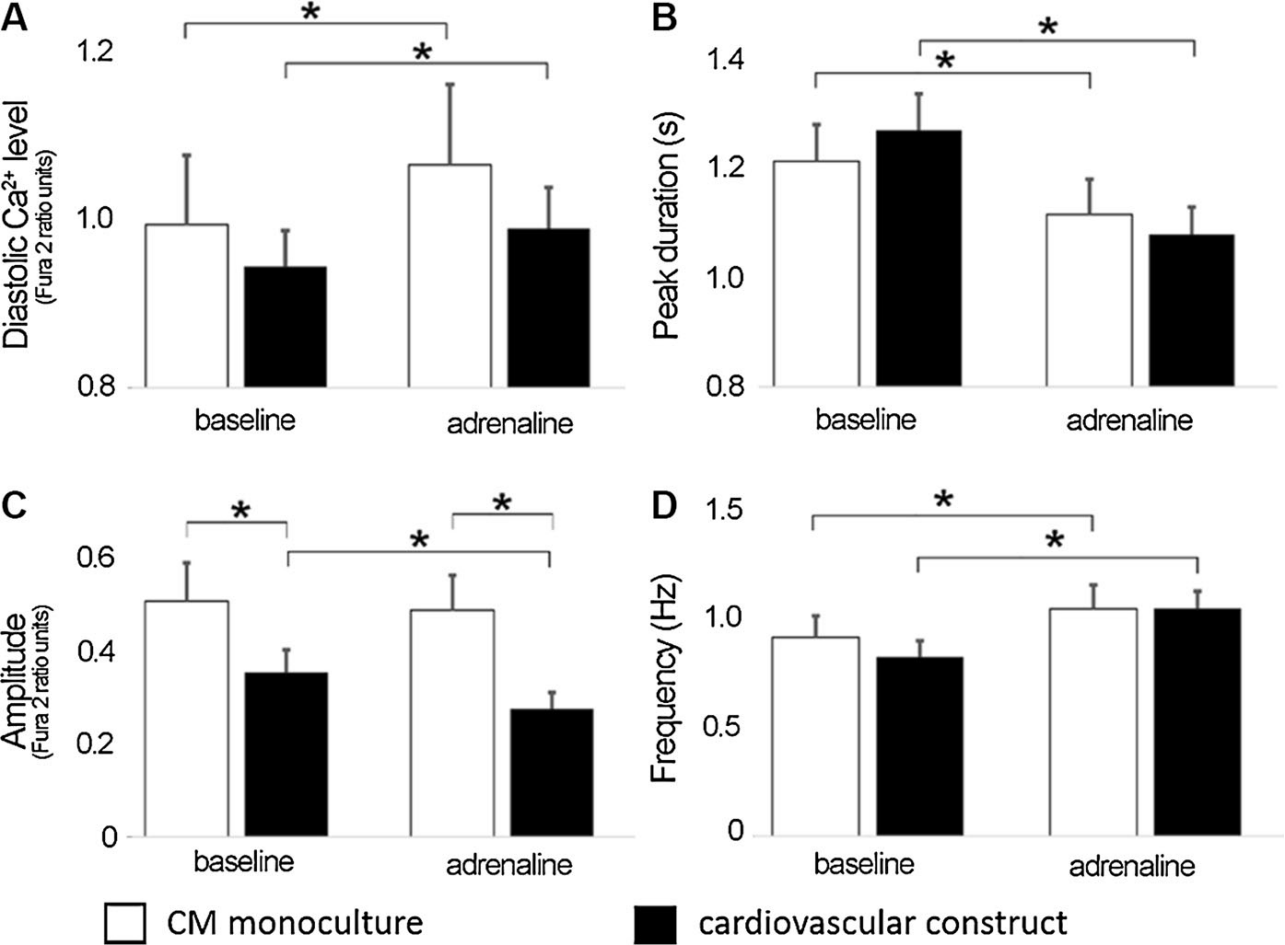
Ca^2+^ measurements of the cardiovascular construct (*black columns*) and cardiomyocyte monoculture (*white columns*) in baseline and after adrenaline exposure represented as **a** diastolic Ca^2+^ levels; **b** peak duration; **c** amplitude and **d** frequency. CM monoculture n = 16, cardiovascular construct n = 26. *p < 0.05

Cardiovascular construct and CM monoculture were shown to respond similarly to adrenaline exposure (See supplemental Fig. S2A). When compared to baseline, adrenaline caused a significant increase in beating frequency (Fig. 6d, *p* < 0.05) and a decrease in peak duration in both systems (Fig. 6b) and, additionally, a significant decrease in amplitude in cardiovascular construct (Fig. 6c, *p* < 0.05). The amplitude in cardiovascular construct was significantly lower at baseline as well as during adrenaline perfusion compared to those in CM monoculture (Fig. 6c, *p* < 0.05).

The diastolic Ca^2+^ increased significantly in CM monoculture and in cardiovascular construct due to the adrenaline exposure (Fig. 6a, *p* < 0.05). Ca^2+^ transient changes were also analyzed separately for each cell as the ratio of adrenaline response divided by baseline response. The results showed that in cardiovascular construct adrenaline caused a significant increase in beating frequency and significant decrease in amplitude when compared to CM monoculture (see supplemental Fig. S2B, *p* < 0.05).

## Discussion

The use of physiologically relevant in vitro model systems is a prerequisite for translating the preclinical data into clinical studies (Engle and Puppala 2013). In preclinical cardiotoxicity studies, the assessment of the risk for QT interval prolongation is part of the standard evaluation of new compounds as defined by the International Conference of Harmonization (ICH) Expert Working Group (Braam et al. 2010). Human pluripotent stem cell derived cardiomyocyte (hPSC-CM) based test systems could improve the early detection of QT prolongation with the benefit of being of human origin (Pfannkuche et al. 2010a). However, it is widely acknowledged that hPSC-CMs possess fetal-like characteristics, e.g. contractility with proliferative capacity and embryonic-like electrophysiology. Although the factors affecting maturity remain largely unknown, cell line, culture time, culture type and other culture conditions appear to have effects on the maturity (Robertson et al. 2013).

We have previously developed cardiovascular constructs based on co-culture of neonatal rat cardiomyocytes and two different vascular-like networks formed either by human adipose stromal cells + HUVECs or HUVECs + fibroblasts. In the previous study, cardiomyocyte viability and functionality was maintained longer in co-cultures and morphological maturation was detected (Vuorenpaa et al. 2014). In the present study, vascular-like network, formed by HUVEC and fibroblasts, was combined with human cardiomyocytes to develop a completely human cell cardiovascular construct for cardiac safety and efficacy assessment and for biomedical research.

Expressions of cardiac structural protein coding genes *MYH6* and *MYH7*, *cardiac troponin T* and gap junction marker *connexin 43* were significantly increased in cardiovascular construct whereas they remained constant or decreased in CM monoculture. In CM monoculture, the overgrowth of non-cardiomyocytes may explain the decreased expression of structural transcripts *MYH6*, *MYH7* and *troponin T*. However, similar overgrowth of non-cardiomyocytes was not detected in cardiovascular construct. Similar results were reported by Caspi et al. (2007) who showed that genes coding structural proteins were expressed at higher level when cardiomyocytes were cultured with endothelial cells and fibroblasts in 3D biodegradable scaffold. Burridge et al. (2014) reported that *troponin T* expression was significantly enhanced at protein level in the multicellular culture when compared to the CM monoculture. Our results are in line with the previous studies, suggesting that vascular-like network improves the cardiac specific phenotype of hPSC-CMs.

In heart, the extracellular matrix, mainly produced by the cardiac fibroblasts, guides cellular orientation and organization thus facilitating efficient cell contraction, force transduction and electrical transmission between the cells. Ventricular cardiomyocytes in the adult human heart are large and brick-shaped whereas hPSC-CMs are markedly smaller with round or triangular morphology (Hartman et al. 2015). The importance of alignment of cardiomyocytes in coordinated contraction is seen in the native cardiac structure, but is also shown in in vitro studies (van Spreeuwel et al. 2014). The alignment of cardiomyocytes has been induced in vitro by microcontact printing, demonstrating that alignment improves cardiomyocyte calcium handling and contractile properties when compared to randomly oriented cardiomyocyte monolayers (Pong et al. 2011; Feinberg et al. 2012). Our results demonstrate that seeding of hPSC-CMs on already formed vascular-like network orientates the hPSC-CMs parallel with the tubule structures. Moreover, as detected in 3D projection, some hPSC-CMs are seen to surround the tubule structures. Fibroblasts are known to produce extracellular matrix components and enhance alignment of cardiomyocytes (Pfannkuche et al. 2010b). The presence of fibroblasts is likely to enhance the orientation of hPSC-CMs with vascular-like structures.

Although earlier studies have shown that co-culture with endothelial cells and fibroblasts enhances the formation of more mature-like morphology of cardiomyocytes and increases the expression of cardiac specific genes and their electromechanical properties (Burridge et al. 2014; Radisic et al. 2008; Caspi et al. 2007), they are based on the utilization of different artificial scaffolds. Since the use of scaffolds in cardiac constructs has been associated with reduced cell–cell contacts, as well as incorrect deposition and alignment of extracellular matrix (Norotte et al. 2009), we aimed at inducing natural in vitro cardiovascular construct. We have previously shown that the vascular-like network formed by HUVEC and fibroblasts serves as a supporting platform for the rat cardiomyocytes (Vuorenpaa et al. 2014). In the present study, vascular-like network serves as a natural, supporting and interactive platform for the hPSC-CMs. Enhanced maturation of hPSC-CM detected already in 12 days suggests that the cardiovascular construct provides a more rapid test system. In addition, we detected increased sensitivity in response to adrenaline, which is likely due to higher expression of the β1-adrenoreceptor in cardiovascular construct compared to CM monoculture.

Our current results with hPSC-CMs showed that the expression of several growth factors including *VEGF*, *FGF-2*, *Ang-1*, *PDGF-β* and *TGF-β* was active in vascular-like network throughout the experiment. In cardiovascular construct, expression levels of growth factors had a similar trend as in the vascular-like network, except for *VEGF* and *PDGF-β* that were increased in the cardiovascular construct. In vascular network, when endothelial cells assemble into tubular structures, endothelial cells start expressing *PDGF-B* to recruit *PDGF-B* receptor positive pericytes to surround the endothelial structures, a step required for tubular maturation. Endothelial cells also secrete *PDGF-B* and other factors in order to modulate cardiomyocytes and assist in the development of heart tissue. As VEGF is mainly produced by the cardiomyocytes (Brutsaert 2003; Leucker et al. 2011), the increase in the expression level of *VEGF *at day 18 is in line with the time in culture. In addition, *Ang-2*, a marker of early stage tubule formation that is expressed when sprouting of tubules and formation of new tubules occurs, was shown to increase in vascular-like network suggesting ongoing tubule formation process in vascular-like network. However, *Ang-2* was low in cardiovascular construct, indicating more mature vascular structures, a result that is also in line with the increased *PDGF-β* expression during culture. Therefore, cardiovascular construct, deprived of exogenous growth factors, was capable of activating the growth factor expression and maintain the differentiation process endogenously.

Our current results suggest that mature vascular-like structures seem not to be the prerequisite for the development of functional cardiovascular construct. More important seems to be the interactive natural stromal support that endothelial cells and fibroblasts provide to the construct. These cells induce cardiomyocytes by secreting several growth factors (Brutsaert 2003; Leucker et al. 2011; Pfannkuche et al. 2010b; Wong et al. 2007) and by providing e.g. endothelial-cardiomyocyte contacts, known to be crucial in maintaining the rhythmic and synchronous contraction of cardiomyocytes (Brutsaert 2003). These cell–cell and cell–matrix interactions as well as paracrine signals of the co-cultured cells, seemed to be critical for the viability of hPSC-CMs and functionality of the developed cardiovascular construct. Although the vascular-like network formation and differentiation cease in the absence of VEGF and FGF-2 during hPSC-CM culture, the cardiomyocyte viability or contractility, as detected in the MEA and calcium metabolism measurements, are enhanced in the presence of the network.

Although the major ionic currents normally present in adult cardiomyocytes are expressed also in hPSC-CMs (Robertson et al. 2013), differences are seen in the expression levels of cardiac ion channels and in calcium handling genes (Synnergren et al. 2007). Our gene expression data showed that in the presence of vascular-like network, the expression of transcripts of sodium (*SCN5A*) and calcium (*CACNA1C*) channels were increased during the days 7–18, while the level of potassium channel (*KCNJ2*) remained constant. More importantly, all these genes were expressed at higher level in the cardiovascular construct compared to CM monoculture at the end of the experiment (day 18). *SCN5A* gene codes the cardiac sodium channel Na_V_1.5 which functions in the fast depolarization phase of the cardiac action potential. The expression level of *SCN5A* has been reported to increase when hPSC-CMs were maturated upon electrical stimulation (Chan et al. 2013). In addition, the expression of *CACNA1C*, gene coding the L-type Ca^+^-channel Cav 1.2, was shown to increase in co-culture with hESC-derived endothelial cells and human amniotic mesenchymal stem cells (Burridge et al. 2014). The expression level of *KCNJ2* that codes the Kir2.1 channel responsible for the inward rectifier potassium current (I_K1_), has been reported to significantly increase during hESC-CM maturation (Sartiani et al. 2007) and in culture with endothelial cells (Burridge et al. 2014). Despite the relative short co-culture time with vascular-like network, cardiomyocytes in cardiovascular construct expressed these ion channel-coding genes at higher level compared to CM monoculture. The increased expression indicates that the aforementioned currents are increasingly present in the cardiovascular construct and the hPSC-CMs would therefore exhibit electrophysiologically more mature phenotype than the monoculture of cardiomyocytes.

MEA measurements showed that E-4031 increased significantly the field potential duration and arrhythmogenicity in the cardiovascular construct as well as in CM monoculture. Calcium imaging analysis showed that Ca^2+^ transients were detectable and adrenaline responses evident in the cardiovascular construct. We detected that adrenaline increased significantly the beating frequency of cardiovascular construct compared to CM monoculture. This was evident in field potential recordings as well as in Ca^2+^ cycling measurements and further confirmed by gene expression analysis. A significantly higher expression of *ADRB1*, a gene encoding the β1-adrenoreceptor, was detected in the cardiovascular construct compared to CM monoculture which further supports the conclusion that vascular-like network has positive effect on the maturation status of the hPSC-CMs.

The specific mechanism how vascular-like network enhances the maturation state of hPSC-CMs remains unknown. Comparative analysis between cardiovascular construct and adult human myocardium could give more detailed information about the maturation state of the construct. However, it can be hypothesized that neighboring non-myocytes could enhance the electrical maturation of hPSC-CMs in cardiovascular construct by secreting paracrine factors or non-cardiac cells might enhance cardiomyocyte signal propagation through cell-to-cell contacts as also suggested by others (Sekine et al. 2008; Narmoneva et al. 2004). Our results suggest that cardiovascular construct presents a more mature in vitro cardiac model compared to CM monoculture and could therefore serve as an advanced test system for cardiac safety and efficacy assessment as well as a model system for biomedical research.

## Electronic supplementary material

Below is the link to the electronic supplementary material.

Supplementary material 1 (DOCX 399 kb)

Supplementary material 2 (AVI 3411 kb)

Supplementary material 3 (AVI 5369 kb)

Supplementary material 4 (AVI 3666 kb)
