# Supplementary material for: Maturation of human pluripotent stem cell derived cardiomyocytes is improved in cardiovascular construct
Source: Cytotechnology. 2017 Apr 10;69(5):785–800. doi: 10.1007/s10616-017-0088-1 (PMC5595750; doi:10.1007/s10616-017-0088-1)
Supplement: Supplementary file 1 — Supplementary material 1 (DOCX 399 kb) [file 10616_2017_88_MOESM1_ESM.docx]

**SUPPLEMENTARY MATERIALS**

**Maturation of Human Pluripotent Stem Cell Derived Cardiomyocytes Is Improved in Cardiovascular Construct**

Hanna Vuorenpää1,3*, Kirsi Penttinen2,3*,#, Tuula Heinonen1,3, Mari Pekkanen-Mattila2,3, Jertta-Riina Sarkanen3,4, Timo Ylikomi 1,3,4 and Katriina Aalto-Setälä2,3,5

1FICAM, Finnish Centre for Alternative Methods, School of Medicine, University of Tampere, Tampere, Finland.

2BioMediTech, University of Tampere, Tampere, Finland.

3School of Medicine, University of Tampere, Tampere, Finland.

4Department of Cell Biology, School of Medicine, University of Tampere, Tampere, Finland.

5Heart Hospital, Tampere University Hospital, Tampere, Finland.

* These two authors equally contributed to this work

# Corresponding author

**Table S1.** SYBR green primer sequences for PCR.

| **Target gene** | **Forward primer** | **Reverse Primer** |
| --- | --- | --- |
| Platelet derived growth factor beta | CTCGATCCGCTCCTTTGATGA | CGTTGGTGCGGTCTATGAG |
| Fibroblast growth factor 2 | AGAAGAGCGACCCTCACATCA | CGGTTAGCACACACTCCTTTG |
| Angiopoietin 1 | AGCGCCGAAGTCCAGAAAAC | TACTCTCACGACAGTTGCCAT |
| Angiopoietin 2 | ACCCCACTGTTGCTAAAGAAGA | CCATCCTCACGTCGCTGAATA |
| Vascular endothelial growth factor | AGGGCAGAATCATCACGAAGT | AGGGTCTCGATTGGATGGCA |
| Transforming growth factor beta 1 | CAATTCCTGGCGATACCTCAG | GCACAACTCCGGTGACATCAA |

**
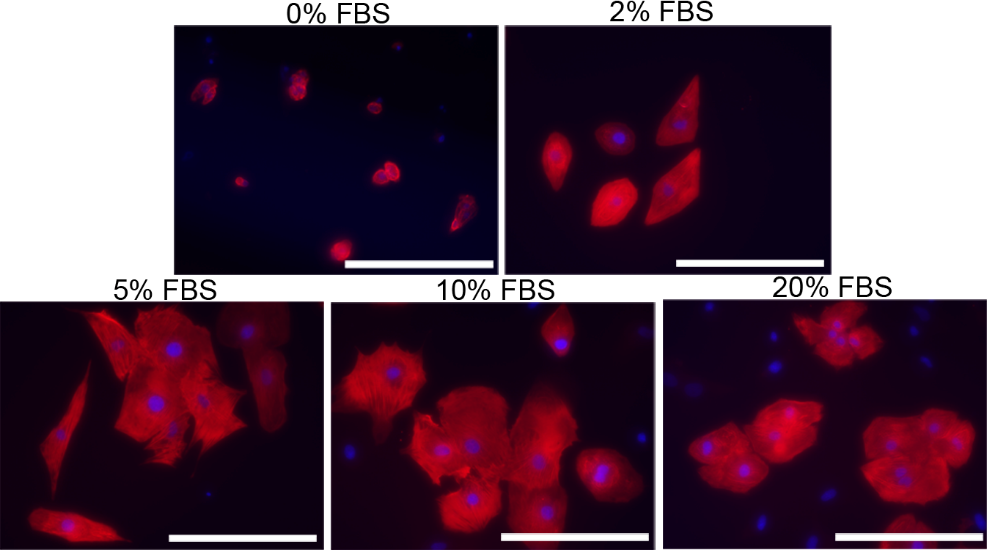


Figure S1.** The effect of fetal bovine serum (FBS) on cardiomyocytes morphology and growth in different concentrations. In the absence of serum, the size of the cardiomyocytes (troponin T, red) was exceptionally small. In the presence of 2 % FBS, the morphology of the cardiomyocytes was still small compared to the cardiomyocytes cultured in 5, 10 or 20 % FBS, in which the cells remained their physiological morphology. Due to our objective to create low-serum culture conditions, the serum concentration for the cardiovascular constructs was chosen to be 5 %. Scale bars 200 µm.

**
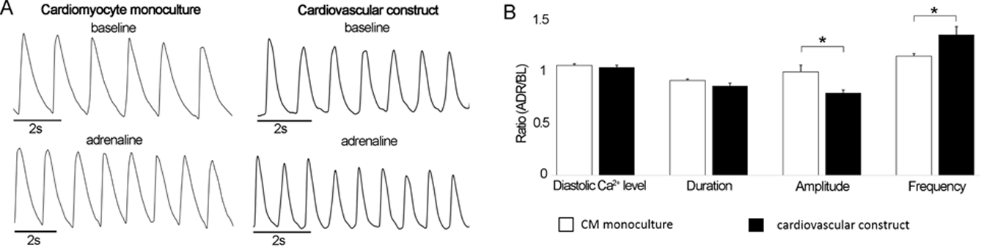
**

**Figure S2.** Calcium cycling of cardiovascular construct and CM monoculture. (a) Representative calcium traces of adrenaline response of CM monoculture and cardiovascular construct. (b) Ratio of adrenalin and baseline measurements of CM monoculture (n=16) and cardiovascular construct (n=26). Values during adrenaline stimulation were divided by values during baseline separately for each cell. The value 1 represents no change between baseline and adrenaline. Error bars: SEM, * p<0.05, a significant difference between cardiomyocyte monoculture and cardiovascular construct.

**Video S1.** An example of the contractility of cardiomyocyte monoculture with 20x magnification with moderately beating separate areas.

**Video S2.** An example of the contractility of cardiovascular model with 20x magnification with synchronous and strong beating.

**Video S3.** An example of the contractility of cardiovascular model with 10x magnification, demonstrating the large size of synchronously beating areas.
